# Limited radiographic detectability of novel 3D printed materials used in dental surgery

**DOI:** 10.1186/s12903-025-07158-w

**Published:** 2025-11-06

**Authors:** Christian Niederau, Rogerio B. Craveiro, Michael Wolf, Philipp Becker, Andreas Pabst, Alexander-N Zeller

**Affiliations:** 1 Department of Orthodontics, Medical Faculty, RWTH Aachen University, Pauwelsstr. 30, Aachen, 52074 Germany; 2 Department of Oral- and Maxillofacial Surgery, Federal Armed Forces Hospital, Rübenacherstr. 170, Koblenz, 56072 Germany; 3 Department of Oral and Maxillofacial Surgery, University Medical Center Mainz, Augustusplatz 2, Mainz, 55131 Germany; 4 Department of Oral- and Maxillofacial Surgery, Hannover Medical School, Carl-Neuberg-Str. 1, Hannover, 30625 Germany; 5 Private Practice for Oral and Maxillofacial Surgery, Theaterstr. 61, Aachen, 52062 Germany

**Keywords:** Foreign body, 3D printing, Insertion or cutting guide, Stereolithography, 3D imaging, Computed tomography (CT), Cone beam computed tomography (CBCT), Fragment, Radiopacity, Digital dentistry

## Abstract

**Objectives:**

The digitalization of numerous dental workflows has significantly expanded the possibilities of digital treatment planning in recent years. By using additively manufactured templates and guides, preoperative planning can be realized reliably and minimally invasively on the patient. However, these devices may break under mechanical stress during use, so that fragments may remain in the surgical field, be swallowed or aspirated. This study investigates the radiologic detectability of additively manufactured materials in surrounding soft tissue.

**Methods:**

The visual detectability of standardized scan bodies and splinters of 15 different materials used in dentistry was analyzed using cone-beam computed tomography (CBCT). Porcine muscle and subcutaneous tissue were used as surrounding structures. In addition, computed tomography (CT) was used to measure the radiation densities of the materials in Hounsfield units to obtain quantitative reference values for the recognizability of the materials in comparison with soft tissues.

**Results:**

The radiodensities in CT images of all modern materials used in computer-aided manufacturing ranged from 68.94 ± 5.32 HU to 130.47 ± 4.52 HU and were thus very similar to those of muscle tissue. In CBCT images, large cylinders as well as small splinters of these materials were hardly visible in both subcutaneous fat and muscular tissues. Overall, the small splinters were more difficult to differentiate from the surrounding tissue than the larger cylinders. The conventional dental materials Futar D^®^ and Luxatemp^®^ exhibited significantly higher radiopacity at 1031.18 ± 12.97 HU and 3243.96 ± 69.03 HU.

**Conclusion:**

The results of this study show that neither CT nor CBCT may currently not be suitable for visualising modern 3D-printed dental materials against surrounding soft tissue. This is due to the materials’ insufficient radiopacity, which prevents clear delineation. Classic materials for temporal direct restorations and silicones were much easier to detect than the tested additively manufactured materials.

**Supplementary Information:**

The online version contains supplementary material available at 10.1186/s12903-025-07158-w.

## Introduction

The ongoing digitalisation in all areas of dentistry has led to a reorganisation of numerous workflows in recent years. As a result, many surgical procedures can be planned three-dimensionally in advance [1]. This planning can then be used during the procedure with the help of an individually manufactured template (e.g. for positioning dental implants [2, 3] or orthodontic temporary anchorage screws [4, 5]). Surgical cutting guides for tooth exposure or orthognathic surgery can also be planned and manufactured with a digital workflow [6, 7]. These techniques reduce treatment time and the risk of side effects [8, 9]. However, there are no guidelines for the production of such templates, so there is a high degree of variability between the designs of different practitioners or dental laboratories. Due to possible under sizing of the material thickness, there is a risk of these templates fracturing during surgery, with the danger of fragments being aspirated, swallowed or left in the surgical area. The search for such fragments is usually carried out with the help of radiological procedures, which, however, require a certain radiopacity of the materials to be able to differentiate them from the surrounding tissue. Insufficient radiopacity of these materials can lead to fragments remaining in the surgical field not being detected at all or only after a delay, which can have negative consequences for the patient’s health and medical law consequences for the practitioner.

New additive manufacturing systems on the market have complemented established systems such as CEREC (Chairside Economical Restoration of Esthetic Ceramics or Ceramic Reconstruction) [10, 11]. In particular, Computer-Aided Design and Computer-Aided Manufacturing (CAD/CAM)-based technologies and additive manufacturing have introduced a variety of new materials into everyday dental practice and surgery [12]. Additive technologies range from advanced (PolyJet printing) and established technologies such as stereolithography (SLA) to entry-level technologies such as fused deposition modeling (FDM) [13–16].

Aspiration, ingestion and other methods of incorporating dental materials and instruments have been the subject of several case reports and reviews [17–20]. Radiological methods such as computed tomography (CT) and cone beam computed tomography (CBCT) are the gold standard for localising these fragments in the maxillofacial region [21–23]. CBCT is characterised by a significantly reduced radiation dose while maintaining high resolution [24] and is therefore usually preferred to conventional CT in the field of dentistry [25]. Furthermore, CBCT is the gold standard imaging technique in implant dentistry [26] and for sinus diagnosis [27]. It is therefore available in many practices and clinics for routine diagnostics and the targeted search for foreign bodies in the oral region and associated surgical sites. It should be noted, however, that the density values measured in Hounsfield Units (HU) as known from CT cannot be transferred to CBCT [28]. Nevertheless, it has been shown that there is a correlation between HU and the grey values of a CBCT, which allows qualitative comparisons between the two types of images [29]. Another important point is that the HU values in CT and the grey values in CBCT depend on the device and the settings used, which can affect the comparability of different measurements [30]. 

Despite the increasing use of novel CAM materials there have been no reports on their radiological properties, to our knowledge. These data are essential for effective CBCT-based foreign body inspection when 3D-printed appliances or their fragments are suspected of being displaced, ingested or aspirated. Therefore, the aim of this study is to evaluate the detectability of critical synthetic materials used in dentistry by CBCT and CT. This will be achieved by examining 15 standardised scan bodies and splinters and evaluating their CBCT visibility and CT radiopacity in comparison to different tissue types, e.g. subcuticular fatty tissue and muscular tissue.

## Materials and methods

### Material selection

Based on the frequency of use in dental laboratory technology, restorative dentistry and dental surgery, 15 materials were selected for the study. The spectrum of materials ranges from established polymethacrylate-based (PMMA) materials (Paladur^®^, Palapress^®^, Paladon^®^ from Kulzer, Hanau, Germany) to new materials on the market, such as polyether ether ketone (PEEK) (KLS Martin, Tuttlingen, Germany), MED610 (Stratasys, Eden Prairie, Minnesota, USA), polyamide (KLS Martin, Tuttlingen, Germany) and 3D-printable acrylic esters (Formlabs^®^, Somerville, Massachusetts, USA) resins, some of which are chemically identical to Nextdent^®^ (Soesterberg, The Netherlands) resins). In addition, materials used on a temporary basis and based on bis-acrylic (Luxatemp^®^ from DMG, Hamburg, Germany) and (Struktur3^®^ from VOCO, Cuxhafen, Germany) and a silicone for bite registration (Futar D^®^ from Kettenbach, Eschenburg, Germany) were included in the study (Table 1).

**Table 1:**
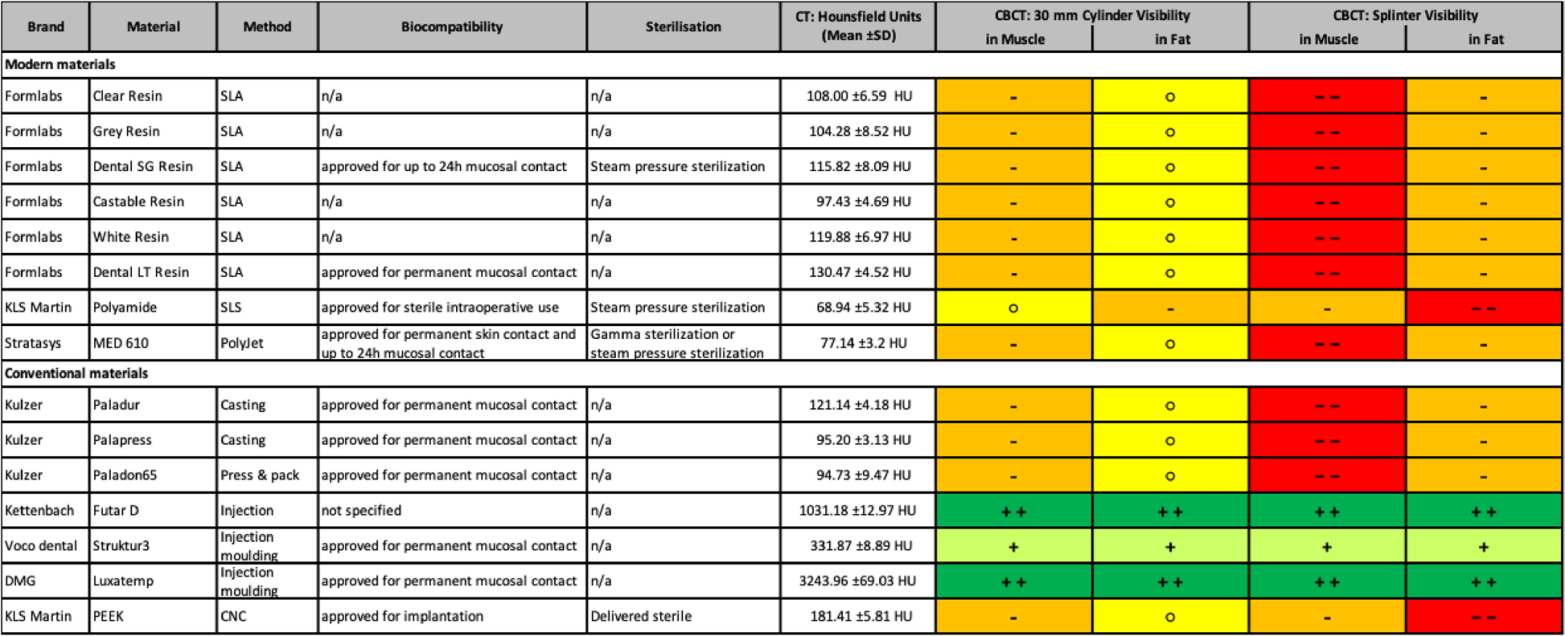
Materials, manufacturers, biocompatibility, available process for sterilization, discriminability against subcuticular fatty and muscular tissues, and corresponding Hounsfield units

### Production of test specimens

**Fig. 1:**
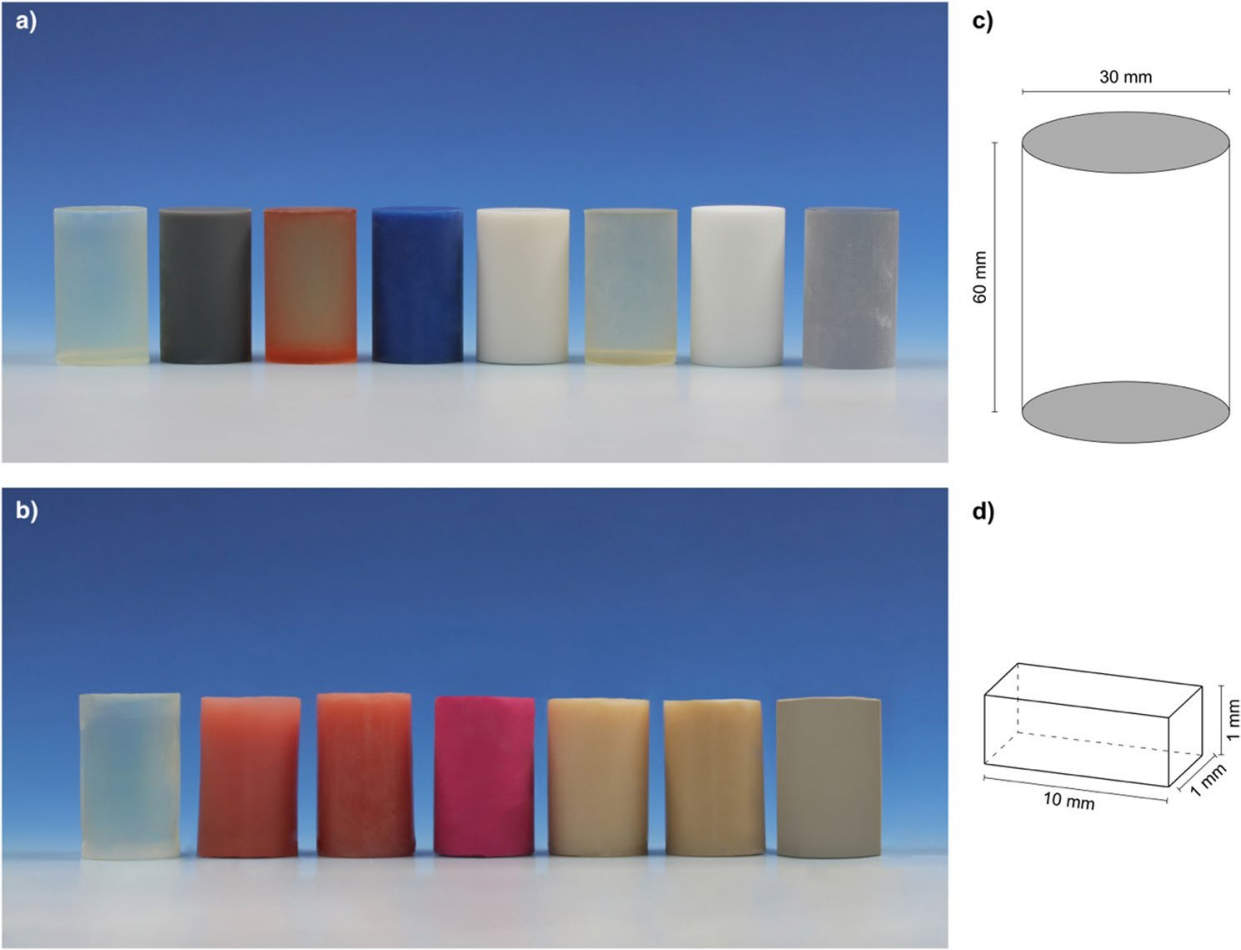
Measuring cylinders composed of the studied materials (descending order as shown in Table 1): **a**) modern materials (Formlabs Clear Resin - Stratasys Med610) and **b**) conventional materials (Kulzer Paladur - KLS Martin PEEK) **c**) dimensions of the cylinders **d**) dimensions of the splinters

To obtain reproducible and comparable data, 30 mm cylinders (Fig. 1) were made from each of the above materials and their diameters were validated using an electronic calliper gauge. Acrylic esters were 3D printed using a Form 2 printer (Formlabs^®^, Somerville, Massachusetts, USA). MED610, polyamide and PEEK cylinders were sourced externally. Following the manufacturer’s instructions, PMMA-based materials were poured into 30 mm hollow cylinders and then water cooled in a pressure pot at 2.2 atm to avoid blistering during curing. Paladon^®^ was packed and pressed into a cuvette filled with hard stone. Three 10 × 1 × 1 mm splinters were cut from each cylinder, and the size was checked using an electronic calliper gauge.

### Examination of visibility in CBCT scans

To assess CBCT visibility, these 30 mm cylinders were placed in a preformed hole in a piece of domestic pig leg (provided by a local butcher) and placed on a polystyrene foam bed. The splinters were pierced into each layer (subcuticular fatty tissue, and muscular tissue). The prepared specimens were then scanned using CBCT (PaX-Zenith3D, VATECH, Hwaseong, South Korea). Scanning was performed in a cylindrical field of view (diameter: 160 mm, height: 140 mm (default setting: Voxel size = 0.2 mm, no metal artefact reduction, 105 kVp, 5.1 mA) and repeated three times.

After CBCT scanning, the axial data of the cylinder and the sagittal reformatted splinter data were exported into VISAGE 7 (Pro Medicus Limited, Richmond, Australia) for further data analysis. With the help of the software, representative sections were selected for each cylinder and saved as screenshots to ensure the best possible comparability. The images were examined by the evaluators on a monitor suitable and tested for radiological diagnosis under constant lighting conditions in the diagnostic room. The visibility of each material against the available tissue (subcuticular fat and muscle tissue) was independently assessed by six craniomaxillofacial surgeons trained in CBCT diagnosis and calibrated for the visibility rating. The evaluators were blinded for the material types. Visibility was rated on an arbitrary scale from “- -” to “+ +” (“--”: Not visible; “-“: Very poorly visible; “o”: Poorly visible; “+”: Well visible; “++”: Very well visible) by examining all screenshots showing the prepared specimens. A visualisation of the visibility ratings is shown in supplementary Fig. 1. After all evaluators had assessed all images independently, a joint consensus decision was reached in the event of disagreement. Data were collected using Microsoft^®^ Excel for Mac (version 16.16.14, Microsoft Corporation, Redmond, USA).

### Determination of CT radiodensity

To determine CT radiopacity, the 30 mm cylinders were placed on a polystyrene foam bed and scanned in a 320-slice CT (Aquilion One, Canon Medical, Ōtawara, Tochigi, Japan) at 120 kV and 450 mAs. Hounsfield units (HU) were measured with the CT system within the cylinders with a volume of interest with a diameter of 20 mm. This volume of interest was positioned at three different locations within the test specimens. The same CT scan was used for each location. Data were collected and averages calculated in Microsoft^®^ Excel for Mac (version 16.16.14, Microsoft Corporation, Redmond, USA).

### Statistical analysis

Fleiss’ kappa was used to examine the results of the evaluation by our six evaluators with regard to inter-rater reliability. Individual values for 30 mm cylinders and splinters were calculated. The calculations were performed in RStudio (Version 2025.09.0) [31].

## Results

All the used cylinders are depicted in Fig. 1; their order corresponds to the descending order in Table 1.

### Interrater reliability of CBCT visibility assessment

Inter-rater agreement among the six raters was assessed using Fleiss’ kappa. For the evaluation of the 30 mm cylinders, agreement was higher (κ = 0.899, 95% CI [0.836–0.961]) compared with the evaluation of the splinters (κ = 0.819, 95% CI [0.759–0.879]). Overall, the inter-rater reliability revealed a high correspondence between the six evaluators.

### Bis-acryl-based materials CBCT visibility

**Fig. 2:**
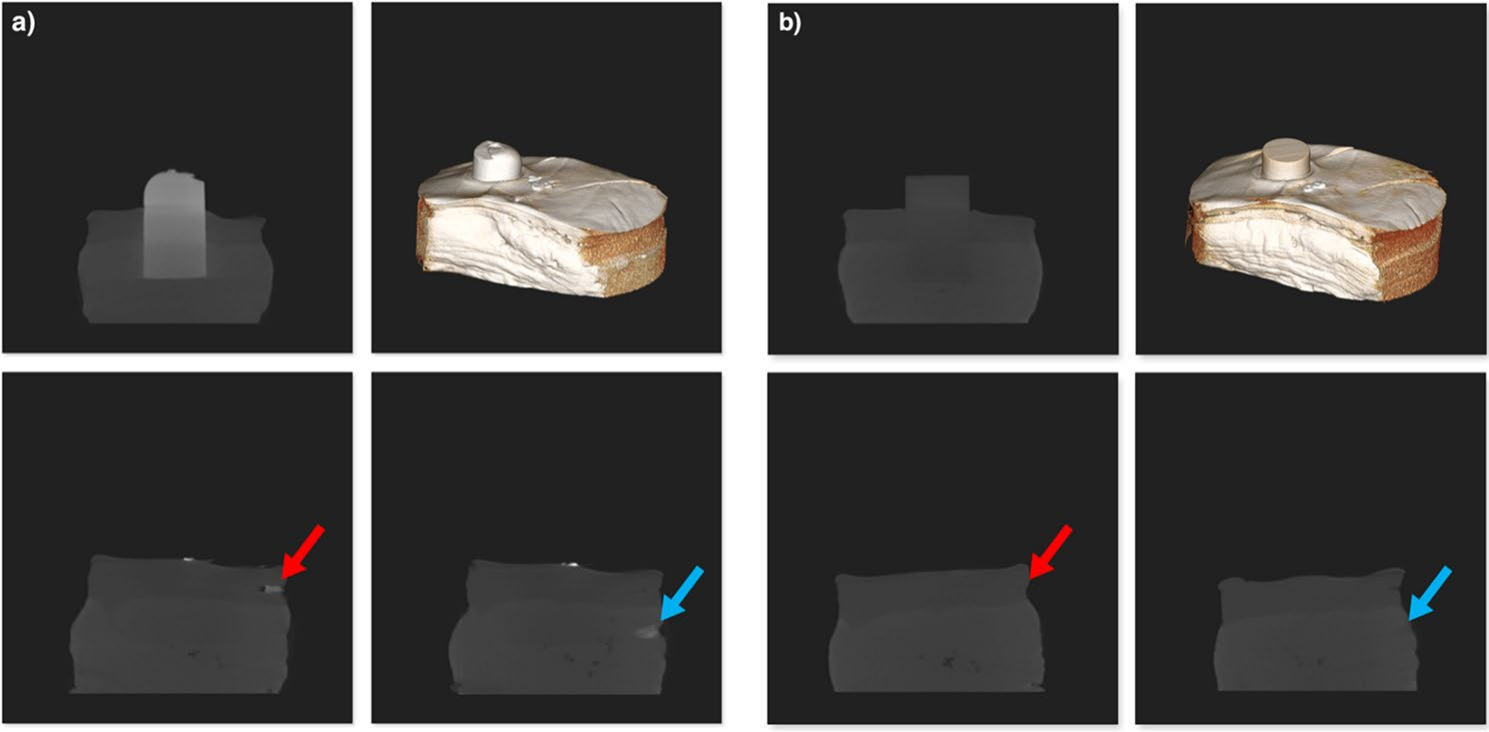
3D rendering of the setup, screenshots of a 30 mm cylinder and splinters in representative layers of the subcuticular fatty tissue (red arrow) and muscular tissue (blue): (**a**) polyamide-based material for additive manufacturing (KLS Martin Polyamide); (**b**) silicone-based material for bite registration (Kettenbach Futar D)

The examined Bis-acryl-based materials for temporal, direct restorations (Voco dental Struktur 3 and DMG Luxatemp), and the bite registration silicone (Fig. 2a, Kettenbach Futar D) exhibited good to excellent visibility in CBCT. This was also true for the splinters of these materials, as indicated in Fig. 2a by the red arrow in the subcuticular adipose tissue and the blue arrow in the muscular tissue. They also presented with high radiodensity in CT, corresponding to Hounsfield units far above those of human tissues (Table 1).

### Acrylic-ester-based materials CBCT visibility

The radiodensities of all the acrylic-ester-based materials (Formlabs^®^ resins: Clear, Gray, Dental SG, Castable, White, and Dental LT), polymethacrylate-based materials (Kulzer Palapress, Paladur, and Paladon65), Stratasys Med610, and PEEK (KLS Martin) were overall almost identical to that of the muscular tissue, resulting in poor visibility of 30 mm cylinders and no visibility of splinters of these materials in this tissue. Visibility in adipose tissue was better than in muscle tissue, but still poorly visible. The larger 30 mm cylinder were better detectable than the splinters. The radiological properties of medical-grade polyamide were comparable to those of the subcuticular fatty tissue.

### Polyamides CBCT visibility

Similar to the materials described before, polyamide splinters, indicated in Fig. 2a by the red arrow in the subcuticular adipose tissue and the blue arrow in the muscular tissue, were difficult to discriminate against the surrounding tissues in comparison with the 30 mm cylinders.

### Radiodensity in Computed Tomography (CT)

**Fig. 3:**
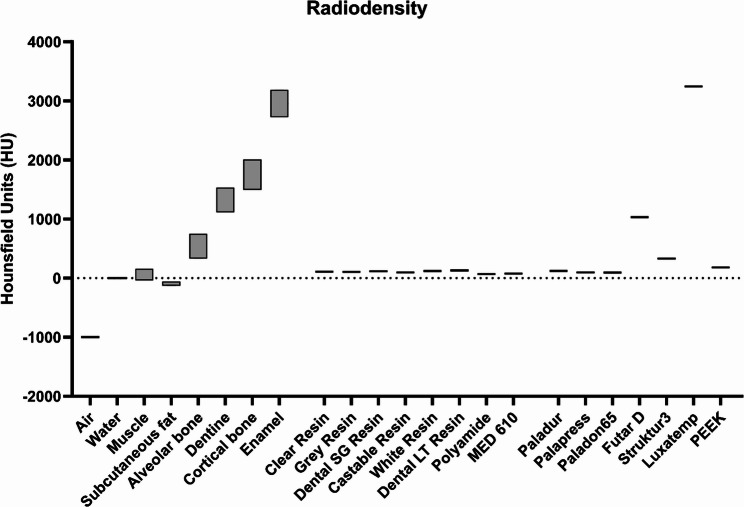
Radiodensities measured in Hounsfield Units (HU) from CT scans of human tissues based on the literature (grey boxes) [35–37, 44, 45] compared to the investigated dental materials

The radiodensity of all 15 materials was determined in CT images with a volume of interest inside the 30 mm cylinders. The measured Hounsfield units of most materials ranged between 68.94 ± 5.32 HU and 130.47 ± 4.52 HU. These values are very similar to those found in human tissues which are relevant for the possible incorporation of material fragments during dental interventions (Fig. 3). Exceptions are the material Struktur3 with 331.87 ± 8.89 HU and the two even more dense materials Futar D (1031.18 ± 12.97 HU) and Luxatemp (3243.96 ± 69.03 HU). These are, as the significantly higher density values compared to human tissue already suggest, also by far the best to identify in the CBCT. The detailed material properties and results are presented in Table 1.

## Discussion

With the development of CAD/CAM technologies, their use in dentistry has increased rapidly. To meet the requirements of biocompatibility, sterilisability and accuracy of the manufactured parts, a variety of new materials have been introduced to the market. Their chemical structure, physical properties and technical processing standards differ significantly from those of traditional materials. The incorporation of any type of dental material and the complications arising from it have been well described in the literature [17–20]. In dental surgery, unlike many other medical disciplines, materials are predisposed to complete or partial incorporation because they are often subjected to high mechanical stress. In addition, materials used for cutting and drilling guides are often in contact with rotating or sharp instruments, and intraoperative modifications are not uncommon. This increases the risk of small fragments becoming detached and remaining in the surgical area. As many 3D-printable materials have not yet been thoroughly evaluated for long-term stability in biological environments, incorporation poses an uncertain risk. Unlike well-established materials such as titanium or ceramics, modern resins may undergo chemical degradation or leach components over time. This could be particularly relevant in cases where material fragments remain unnoticed in the surgical site.

When such material is suspected to have been incorporated, either in whole or in part, foreign body examination should be facilitated by excellent detectability using standard techniques. A review of the literature suggests that not only CT but also CBCT is in general a viable examination method for foreign body detection [32–34]. However, the results presented in this study show that CT and CBCT may be inadequate for reliably detecting these modern 3D-printed materials. The radiographic visibility in CBCT and CT images of almost all the materials tested was generally poor. Conventional polymethacrylate-based materials and all the modern materials tested were barely visible in the subcuticular fat and muscle tissues. Small fragments were generally less easy to identify than larger samples. The CT-based Hounsfield units of these materials were in the range of those physiologically present in human tissues [35–37]. Discrimination of these materials during radiographic foreign body examination is therefore likely to be very challenging. Besides the health risks coming with it, limited detectability of material fragments may also have forensic and medicolegal implications. In postoperative diagnostics or legal disputes, poor visibility complicates documentation and the identification of potential complications. This could hinder the assessment of treatment outcomes and accountability in the event of adverse incidents.

The problem of limited visibility of certain traditional dental restorative materials is well known and attempts have been made to overcome this problem by adding radiopaque elements [38]. The atomic number, also known as nuclear charge, is relevant for selecting the element. The higher the atomic number (Z), the greater the absorption capacity of X-rays by the material and thus also the radio-opacity [38]. Therefore, elements like barium (*Z* = 56), yttrium (*Z* = 39), strontium (*Z* = 38) are suitable to enhance radio-opacity. The use of such additives to improve X-ray opacity has been standard practice in numerous medical and dental applications for many years [39, 40]. However, this has not yet been the case for 3D-printable materials. One study demonstrated the potential of adding triphenyl bismuth (TPB) (Z = 83) to dental acrylics [41]. Although there were no significant changes in the physical properties, this method has not been widely accepted. The radiopacity of modern acrylic esters for SLA has been shown to be significantly improved by the addition of small amounts of barium sulphate [42] without compromising print accuracy. However, further research is needed to optimize the composition of 3D-printed materials with regards to their radiopacity. It should be noted that the radiopacity of dental materials should be adjusted to a reasonable level. Excessive contrast formation can lead to the appearance of artefacts in X-ray images [43], which is particularly relevant if the appliances made from the material must remain in the mouth during the exposure (e.g. splints for orthognathic surgery). Although several radiopaque fillers are widely used in medical and dental devices, their adaptation for printable resins still needs targeted evaluation. Therefore, their clinical application appears to be experimental at present and requires further research. Besides that, effects of printing parameters such as infill density, layer thickness, and printing orientation may have an influence on the radiographic visibility of 3D-printed materials. These factors may influence the physical density and internal structure of the material, which in turn affects its detectability on radiographic imaging.

This study has certain limitations that should be acknowledged. The analysis was conducted under standardized 3D-printing conditions, without exploring the influence of variable layer thicknesses, build orientations, or differing infill strategies. These parameters may alter the internal structure and surface characteristics of the appliances, potentially impacting their detectability in 3D radiographic imaging. Moreover, the study did not investigate different post-processing procedures such as UV curing or other hardening protocols commonly applied to enhance the mechanical stability of printed resins. Such processes should be included in future research to better reflect individual clinical workflows.

Manufacturers of 3D-printable dental materials for intraoral use should be aware of the need for detectability in clinical imaging and promote the development of detectable compositions. Furthermore, information on material composition and detectability should be provided, for example in the material’s safety data sheet, so that the practitioner can immediately select and use the best possible method of identification. In addition, a publicly accessible database could be created to provide such information without the need for time-consuming searches. This could also include other possible detection methods such as MRI or sonography. These alternative imaging techniques may provide further opportunities for detecting fragments of 3D-printed devices. A major advantage of these methods would be the elimination of X-rays. However, the necessary equipment is often not available in oral surgery practices, which is why spontaneous use directly during the procedure is not possible. It can be discussed whether this should be a regulatory requirement, or not. Given the increasing use of 3D-printed materials in clinical settings, regulatory bodies such as the FDA or the European MDR may warrant standards for radiographic detectability and safety. Currently, no uniform requirements exist for the imaging visibility of intraoral or surgical materials, which may pose a challenge for clinical implementation and quality assurance.

## Conclusion

The CBCT visibility and CT radiodensity of the additively manufactured non-metallic materials currently used in dentistry were generally inadequate among the materials tested. The radiopacity of such materials is very similar to that of soft tissue and is therefore insufficient for reliable detection by CT and CBCT. Therefore, in order to improve patient safety, the addition of radiopaque minerals such as TPB or barium sulphate to modern materials should be considered to improve their radiological visibility.

The poor radiological detectability of additively manufactured materials currently means that dental professionals must design devices made from them with particular attention to ensuring that the material is thick enough to minimise the risk of breakage. Care should also be taken to reduce the mechanical stress on these parts during intra-operative use.

## Supplementary Information

Supplementary Material 1.

## Data Availability

The datasets used and analysed during the current study are available from the corresponding author on reasonable request.
