# Supplementary material for: Limited radiographic detectability of novel 3D printed materials used in dental surgery
Source: BMC Oral Health. 2025 Nov 6;25:1758. doi: 10.1186/s12903-025-07158-w (PMC12593903; doi:10.1186/s12903-025-07158-w)

Supplementary Figure 1: Schematic overview of the visibility ratings of 3D-printed dental materials in CBCT scans.

The ratings correspond to those in Table 1. Please note that this figure is for illustrative purposes only. The appearance may be affected by the monitor used for viewing, its settings, and calibration.

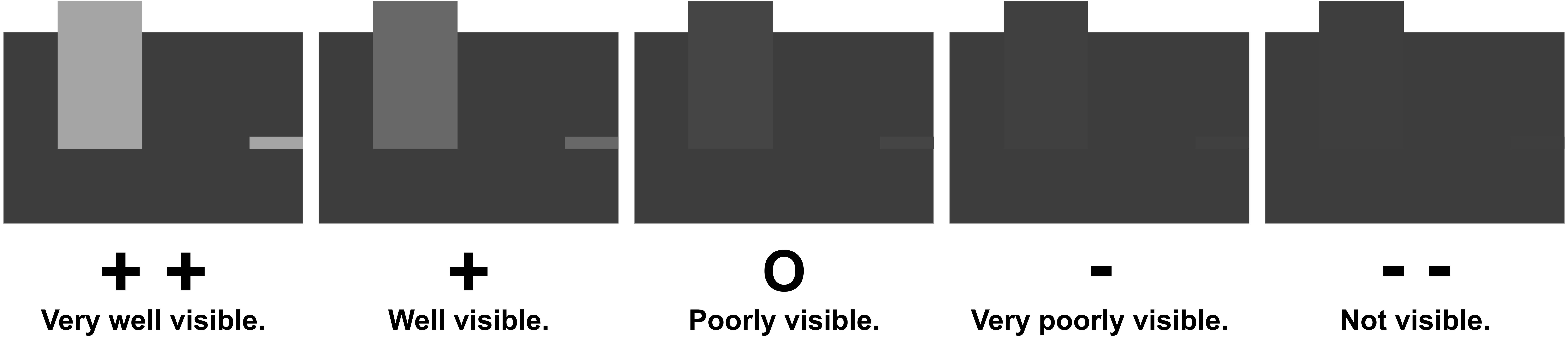

Supplement: Supplementary file 1 — Supplementary Material 1. [file 12903_2025_7158_MOESM1_ESM.pdf]
